# Mr. Urvins, on Quackery

**Published:** 1800-06

**Authors:** David Urvins

**Affiliations:** Grenville Street, London


### *To the Editors of the Medical and Phyfical Journal.*

GENTLEMEN,

SHOULD the following obfervations meet with your approbation, by devoting a page of your highly ufeful and extenfively circulated publication to the admiffion of them, you will confer an additional obligation on

Your obedient fervant,

*Grenville Street, London.* DAVID URVINS.

*May* 5, 1800.

---

Two gentlemen, a few months fince, took occafion, through the medium of the Medical and Phyfical Journal, to lament the alarming progrefs which *empiricifm*, in different forms, is making in this and in other countries; and, at the fame time, propofed means which, to them, appeared moft eligible for its fuppreffion and abolifhment.

After the fubject had been for fome time in a ftate of dormancy, I was extremely happy in obferving, in the table of contents of this excellent publication, a paper entitled, "*On the pernicious Effects of Quackery*," hoping therein to meet with fome obfervations more confonant with my own ideas, refpecting the method which ought to be purfued, for the purpofe of arrefting this lamentable evil.

Upon perufing this paper, I, however, fuffered a degree of difappointment, in finding no additional matter to what had been before advanced on the fubject, excepting the publication of a few cafes, which were by no means neceffary to ftrengthen the evidence againft all kinds of noftrums and fpecifics.

Without wifhing in the leaft to detract from the merit, ingenuity, and philanthropic intentions of thofe gentlemen who have favoured the public with their opinions on the fubject; I beg leave to obferve, that in their zeal for the extermination of quackery, they have neglected to trace it to its origin and foundation.

To what are we to attribute the confidence which is fo generally placed in the falfe atteftations and hyperbolical affertions of men, who are only recommended to public attention by the extremes of *ignorance*, *temerity*, and *effrontery*; while thofe of *character*, *talents*, and *judgment*, in the medical profeffion, are frequently fuffered to remain in the utmoft obfcurity, neglected and difefteemed?

I have

I have no hesitation in affirming, that *ignorance on the part of the public*, and not neglect of legislative interference, is the grand basis on which quackery is founded.

The benevolent exertions and persevering industry of those gentlemen who are endeavouring to introduce the physiological part of the medical science to public attention, cannot therefore, be too highly esteemed or commended.

There is a class of individuals, which form a large part of the medical practitioners of this country, who, without education, without abilities; nay, sometimes, even destitute of common sense, having, perhaps, for a few years been in the habit of vending and compounding medicines in the shop of a druggist or an apothecary, have the effrontery to deem themselves qualified to practise physic, and enroll themselves in the list of medical professors!

Such characters as these, being permitted without detection to pursue their "murderous career," is an evil of still greater magnitude, and calls more loudly for the attention of medical men, than the more open profession and practice of the publishers of nostrums and universal remedies.

The dissemination of medical, or rather physiological knowledge, would enable unprofessional individuals to distinguish the scientific and meritorious from the bold and ignorant class of men above described; would infallibly destroy the blind confidence which has hitherto been reposed in quacks, nostrums, and specifics; and would, consequently, prove of the greatest advantage to the regular practitioners of the important science of medicine.

Happy will it prove for society, when, in consequence of the philanthropic exertions of *Dr. Beddoes* and others, ignorance and temerity will be banished from physic, when quacks and specifics shall no longer exist!

